# The TNFR1 Antagonist Atrosimab Is Therapeutic in Mouse Models of Acute and Chronic Inflammation

**DOI:** 10.3389/fimmu.2021.705485

**Published:** 2021-07-07

**Authors:** Fabian Richter, Sarah K. Williams, Katharina John, Carina Huber, Camille Vaslin, Henri Zanker, Richard Fairless, Kira Pichi, Silke Marhenke, Arndt Vogel, Marie-Ann Dhaen, Stefanie Herrmann, Andreas Herrmann, Klaus Pfizenmaier, Heike Bantel, Ricarda Diem, Roland E. Kontermann, Roman Fischer

**Affiliations:** ^1^ Institute of Cell Biology and Immunology, University of Stuttgart, Stuttgart, Germany; ^2^ Stuttgart Research Center Systems Biology, University of Stuttgart, Stuttgart, Germany; ^3^ Department of Neurology, University Clinic Heidelberg, Heidelberg, Germany; ^4^ Clinical Cooperation Unit (CCU) Neurooncology, German Cancer Consortium Deutsches Konsortium für Translationale Krebsforschung (DKTK), German Cancer Research Center Deutsche Krebsforschungszentrum (DFKZ), Heidelberg, Germany; ^5^ Department of Gastroenterology, Hepatology and Endocrinology, Hannover Medical School, Hannover, Germany; ^6^ Baliopharm, Basel, Switzerland

**Keywords:** TNF, TNFR1, inflammatory diseases, arthritis, EAE, multiple sclerosis

## Abstract

Therapeutics that block tumor necrosis factor (TNF), and thus activation of TNF receptor 1 (TNFR1) and TNFR2, are clinically used to treat inflammatory diseases such as rheumatoid arthritis, inflammatory bowel disease and psoriasis. However, TNFR1 and TNFR2 work antithetically to balance immune responses involved in inflammatory diseases. In particular, TNFR1 promotes inflammation and tissue degeneration, whereas TNFR2 contributes to immune modulation and tissue regeneration. We, therefore, have developed the monovalent antagonistic anti-TNFR1 antibody derivative Atrosimab to selectively block TNFR1 signaling, while leaving TNFR2 signaling unaffected. Here, we describe that Atrosimab is highly stable at different storage temperatures and demonstrate its therapeutic efficacy in mouse models of acute and chronic inflammation, including experimental arthritis, non-alcoholic steatohepatitis (NASH) and experimental autoimmune encephalomyelitis (EAE). Our data support the hypothesis that it is sufficient to block TNFR1 signaling, while leaving immune modulatory and regenerative responses *via* TNFR2 intact, to induce therapeutic effects. Collectively, we demonstrate the therapeutic potential of the human TNFR1 antagonist Atrosimab for treatment of chronic inflammatory diseases.

## Introduction

Tumor necrosis factor (TNF) is recognized as a master pro-inflammatory cytokine that plays a dominant role in the initiation and perpetuation of chronic inflammation (1, 2). Elevated levels of TNF are observed in autoimmune and degenerative diseases and deregulated TNF expression and signaling has been implicated in the pathology of many inflammatory diseases (3, 4). Accordingly, therapeutics that specifically neutralize the activity of TNF were developed. Currently, five anti-TNF therapeutics and additional biosimilars thereof are approved and successfully used to treat autoimmune diseases, including rheumatoid arthritis (RA), juvenile RA, inflammatory bowel disease, psoriasis, and ankylosing spondylitis (4). Despite their clinical success, these anti-TNF therapeutics have limitations. Depending on the anti-TNF therapeutic used, approx. 30% of Crohn’s patients do not respond to the treatment and up to 46% stop responding during therapy (5). Furthermore, anti-TNF drugs can lead to severe side-effects, including common and opportunistic infections, reactivation of latent tuberculosis, increased susceptibility for development of additional autoimmune diseases, demyelination diseases, and an increased risk to develop lymphomas (3, 4). Importantly, clinical evaluation of an anti-TNF therapy in multiple sclerosis (MS) failed (6, 7) and anti-TNF therapy of juvenile rheumatoid arthritis resulted in development of MS-like exacerbations and demyelinating lesions in some patients (8).

The side-effects of anti-TNF therapeutics and the failed MS trials can be explained by the pleiotropic, often opposing TNF responses mediated through the two transmembrane TNF receptors, TNF receptor 1 (TNFR1) and TNFR2. It was shown that expression levels of TNFR1 and TNFR2 are regulated during inflammatory conditions and that cellular fate may be dependent on cytokine-mediated TNFR expression (9). Whereas TNFR1, which is activated by the membrane-bound (tmTNF) and soluble (sTNF) form of TNF, promotes cell death and inflammation, TNFR2 is only robustly activated by tmTNF and contributes to tissue regeneration and immunomodulation (10). We previously have confirmed these differential effects using the experimental autoimmune encephalomyelitis (EAE) animal model of multiple sclerosis (11). Here, we showed that mice lacking TNFR1 develop a dramatically ameliorated disease course, whereas TNFR2-deficient mice show more severe signs of EAE motor disease. Therefore, global inhibition of both sTNF and tmTNF by the approved conventional anti-TNF therapeutics may be counterproductive in certain diseases, such as MS. Consequently, next generation therapeutics have been developed that either neutralize sTNF (12, 13) or directly inhibit TNFR1 activation (14, 15). In particular, we developed Atrosab, a monoclonal antagonistic humanized TNFR1-specific antibody (14, 16). Atrosab demonstrated therapeutic activity in the EAE model of MS (17) and a model of non-alcoholic fatty liver disease (18). However, a first clinical phase 1 study with Atrosab revealed dose-limiting side effects at rather low doses (10). This was attributed to a marginal agonistic response in a small concentration range observed *in vitro* due to bivalent TNFR1 binding of the full IgG molecule (14). Thus, we next developed Atrosimab, an optimized monovalent derivative of Atrosab, composed of affinity improved VH and VL domains fused to an effector-silenced heterodimerizing Fc region (19). *In vitro*, Atrosimab showed superior blocking activity compared to the parental bivalent molecule Atrosab (19). In this study, we validated the *in vitro* stability of Atrosimab and confirmed its therapeutic activity in mouse models of inflammatory diseases.

## Material and Methods

### Materials

Atrosab, Atrosimab, FcΔab and human TNFR1-Fc were provided by Baliopharm (Basel, Switzerland). Mouse, rat, rhesus and cynomolgus TNFR1-Fc and human and mouse TNFR2-Fc were obtained from Sino Biologicals (Eschborn, Germany). Atrosimab was produced by Catalent (Catalent Pharma Solutions, Madison, Wisconsin, US) in chinese hamster ovary (CHO) cells after lentiviral transfection and purified by depth filtration (Zeta Plus™), anion exchange hybrid purifier (Emphaze AEX filter), protein A affinity (Amsphere™ A3, bind-elute mode), anion exchange (Toyopearl^®^ NH2-750F, flow-through mode) and CHT multi modal (CHT Type I, bind-elute mode) chromatography. Virus inactivation was performed by incubation of Amsphere load with Triton X-100 and virus removal by Nanofiltration (Virosart^®^) of CHT eluate.

### Protein Characterization

Atrosimab was analyzed by sodium dodecyl sulphate–polyacrylamide gel electrophoresis (SDS-PAGE) using 4 μg protein under reducing and non-reducing conditions. Proteins were stained using Coomassie-Brilliant Blue G-250 and acrylamide gels were de-stained with water. Correct assembly under native conditions was visualized by size exclusion chromatography (SEC) using a Waters 2695 HPLC and a TSKgel SuperSW mAb HR column (flow rate of 0.5 ml/min, 0.1 M Na_2_HPO_4_/NaH_2_PO_4_, pH 6.7 as mobile phase). Standard proteins (MW, SR) included Thyroglobulin (669 kDa, 8.50 nm), Apoferritin (443 kDa, 6.10 nm), beta Amylase (200 kDa, 5.4 nm), bovine serum albumin (67 kDa, 3.55 nm), and Carbonic anhydrase (29 kDa, 2.35).

### Protein Stability Assays

Temperature-dependent aggregation of Atrosimab was analyzed by dynamic light scattering using the ZetaSizer Nano ZS (Malvern, Herrenberg, Germany). Atrosimab (100 µg in PBS) was heated stepwise from 35°C to 80°C with intervals of 1°C and equilibration times of 2 min prior to each measurement and signal of the particle size (kcps) was determined. The mean of two measured kcps values at each temperature was calculated and plotted over temperature. The aggregation temperature was defined as the temperature T where the quotient kcps_T_/kcps_(T-5)_ reached at least a factor 2.0. Melting points were further determined by differential scanning calorimetry in the laboratory of Prof. Franz Hagn (TU Munich, Germany).

### Enzyme-Linked Immunosorbent Assay for Binding Studies

The indicated fusion proteins were diluted to 1 μg/ml in PBS, transferred to a 96-well microtiter plate (Greiner, microlon) and incubated overnight at 4°C. Skim milk in PBS (2% MPBS) was used to block residual binding sites [200 μl/well for 2 h at room temperature (RT)]. Atrosimab or EHD2-sc-mTNF_R2_ (20) was diluted in 2% MPBS to a maximal concentration of 500 nM and diluted in steps of 1 to 3.16 (square root of 10). Each sample was transferred to the microtiter plates and incubated at RT for 1 hour. Anti-human IgG (Fab-specific)-HRP labeled detection antibodies (Sigma A0293) were diluted in 2% MPBS (1:20,000) and incubated for 1 h at RT. Binding of Atrosimab was detected using 100 μl 3,3ʹ,5,5ʹ-Tetramethylbenzidine (TMB) substrate solution. The enzymatic reaction was stopped using 50 μl 1 M H_2_SO_4_. Absorption was determined at a wavelength of 450 nm. In each step, a working volume of 100 μl was used and between each step, the plates were washed twice with PBS containing 0.05% Tween-20 (PBST) and twice with PBS.

### IL-8 Release Assay

7,500 HT1080 cells were plated in 100 μl RPMI 1640 supplemented with 5% FCS in 96-well plates and incubated at 37°C, 5% CO_2_ overnight. Then, the medium was removed, Atrosimab samples were diluted in steps of 1 to 3.16 (square root of 10) in culture medium and applied to the cells. In the case of inhibition experiments (antagonist assay), addition of Atrosimab samples was followed by addition of TNF (0.01 nM). For agonist assays, cells were incubated with Atrosimab dilution, but without addition of TNF. After 16 - 20 h incubation at 37°C, 5% CO_2_, the supernatants were harvested subsequent to 5 min centrifugation at 500 *g* and analyzed for IL-8 concentration by ELISA according to the manufacturer’s instructions (ImmunoTools, Friesoythe, Germany).

### Affinity Measurements

The affinity measurements were performed by SPR technology using a Biacore T200. Cynomolgus and human TNFR1 was immobilized on a CM5 (carboxymethylated Dextran matrix) chip surface (one flow cell each with remaining flow cells being blanks) using standard amine coupling chemistry (10 mM Sodium Acetate pH 4.0). Subsequently Atrosimab was serially diluted in assay buffer (HBS-EP+, Hepes buffered saline with EDTA and P20) from 500 nM to 2 nM (2-fold dilution) and injected over each flow cell pair in separate assay sections (multi cycle kinetic), the regeneration was performed with 3M MgCl_2_. The data generated were fitted to a 1:1 binding model.

### Flow Cytometry of Endothelial Cells

hCMEC/D3 cells (21) were cultured and stained as described previously (Williams et al., 2018). Briefly, cells were stimulated for 24 hours with human TNF (100 ng/ml, R&D Systems, Minneapolis, MN, USA) in presence or absence of Atrosimab (100 µg/ml). Then cells were harvested and collected in PBS containing 0.02 mM EDTA and 0.4 mg/ml collagenase (Sigma-Aldrich). Cells were then stained with antibodies against either human VCAM-1 (CD106-PE, STA, eBioscience) or ICAM-1 (CD54-APC, HA58, eBioscience). Cells were then fixed in 1% PFA and kept in the dark until analysis. Flow cytometry acquisition was performed on a FACSCanto II (BD Biosciences) using the BD FACSDiva software. All data was analyzed using FlowJo software.

### Mice

All animal studies were performed according to the EU guidelines and have been approved by University authorities and local governmental institutions (Stuttgart, Heidelberg, Hannover). The huTNFR1-k/i mice were described previously (22). Briefly, these are humanized transgenic animals, where the endogenous TNFR1 is exchanged for a chimeric TNFR1 composed of the extracellular domains of human TNFR1 fused to the transmembrane and intracellular part of mouse TNFR1. Tg197hTNFR1KI is a double humanized transgenic mouse model that expresses human TNF and human TNFR1. These mice were generated by crossing Tg197 mice with huTNFR1-k/i mice. Tg197 mice overexpress human TNF and develop spontaneously chronic arthritis pathology closely resembling human rheumatoid arthritis (23).

### Pharmacokinetics

Atrosimab and Atrosab were injected at doses of 1.0 mg/kg or 30.0 mg/kg body weight intravenously (i.v.) or subcutaneously (s.c.) in C57BL/6J wild type mice or C57BL/6J huTNFR1-k/i mice. In transgenic huTNFR1-k/i mice the endogenous mouse TNFR1 is exchanged with a chimeric TNFR1, composed of the extracellular domain of human TNFR1 fused to the mouse transmembrane and intracellular region (22). Blood samples were collected at the indicated time points and incubated on ice for 20 min. Serum was separated by centrifugation (13,000 *g*, 4°C, 10 min) and stored at −20°C prior to analysis. The concentration of Atrosimab in serum samples was determined by an ELISA specific for Atrosimab as described for the binding studies. Results were analyzed using the PKSolver Excel add-in (i.v. studies) or GraphPad Prism One-Phase decay non-linear fitting algorithm (s.c.).

### Safety Study

Mice received i.v. injections of 100-150 µl Atrosimab (30.0 mg/kg), FcΔab (30.0 mg/kg) or TNF (0.3 mg/kg), or were left untreated, respectively. Body weight was recorded at the indicated time points. Blood samples were collected at the indicated time points, incubated on ice for 20 min and serum was separated by centrifugation (13,000 *g*, 4°C, 10 min). Serum samples were stored at -20°C subsequently. At the end of the study, serum samples were analyzed for IL-6 (IL-6 Duo-Set, R&D, DY406) and CRP levels (CRP Duo-Set, R&D, DY1829) according to the instructions of the manufacturer. Individual values were excluded using the ROUT method of outlier identification (Q=1%).

### TNF-Mediated IL-6 Release *In Vivo*

Mice first received an i.v. injection (100-150 µl) of Atrosimab (30.0 mg/kg), FcΔab (30.0 mg/kg), or PBS, respectively, followed 30 minutes later by i.v. injection of TNF (0.3 mg/kg). Body weight was recorded at the indicated time points. Blood samples were collected at the indicated time points, incubated on ice for 20 min and serum was separated by centrifugation (13,000 *g*, 4°C, 10 min). Serum samples were stored at -20°C subsequently. At the end of the study, serum samples were analyzed for IL-6 (IL-6 Duo-Set, R&D, DY406) according to the manufacturer’s instructions. Individual values were excluded using the ROUT method of outlier identification (Q=1%).

### Experimental Arthritis

Weight of Tg197hTNFR1KI mice was recorded weekly on day of arthritis scoring. Arthritic disease was scored using a scale ranging from 0 to 3: 0, no arthritic disease; 0.5, mild joint swelling; 1.0, joint distortion by swelling, inflamed paw; 1.5, joint-paw swelling. distortion + last finger inward deformation, brief support clinging to an inverted or tilted surface such as a wire grid or a cage lid, whole body flexibility reduced, reduced grip strength; 2.0, severe joint, paw and finger swelling, joint - leg deformation, no support clinging to an inverted or tilted surface such as a wire grid or a cage lid, no whole-body flexibility, no grip strength, climbing/feeding affected, starts shaking when trying to move, but manages to move forward; 2.5, as above 2 + finger deformation in front paws, mouse movement impaired, shaking not willing to move; 3.0, ankylosis detected on flexion and severely impaired movement, mouse moribund, not shaking anymore, cannot turn/flip around readily when tilted to the side. Mice were treated with Atrosimab or anti-TNF therapeutics as described in the figure’s legend.

### Induction of Non-Alcoholic Steatohepatitis

Non-alcoholic steatohepatitis (NASH) was induced in male huTNFR1-k/i mice by feeding a high-fat diet (HFD) consisting of 60% kcal of fat (Altromin, Lage, Germany), supplemented with 42 g/L sugar (55% fructose, 45% sucrose) in the drinking water (18) for 21 weeks, before therapeutic intervention was initiated at week 22 by administration (i.p.) of Atrosab or Atrosimab (45 mg/kg body weight, 2×/week) for further 8 weeks. Saline treatment was used as a treatment control. Mice were randomly distributed to the different treatment groups. Mice of both groups revealed no significant difference in age. At the end of treatment, mice were sacrificed, liver weight was determined, and liver tissues were prepared for immunohistological assessment of fibrosis, fat content and caspase activation.

### Induction and Analysis of Experimental Autoimmune Encephalomyelitis

EAE was induced as previously described (11, 17). Briefly, female mice (6,7,8 weeks old) were immunized s.c. with 300 μg MOG^35−55^ in PBS emulsified in an equal volume of complete Freund’s adjuvant (Sigma-Aldrich Corp., St. Louis, MO) supplemented with Mycobacterium tuberculosis H37RA (Difco Laboratories Inc., Detroit, MI) to a final concentration of 10 mg/ml. Immediately afterwards and again 2 days later 300 ng pertussis toxin was injected intraperitoneally (i.p.). Mice were weighed and scored on a daily basis, with disease severity assessed using a scale ranging from 0 to 5: 0, no clinical disease; 0.5, distal paresis of the tail; 1.0, complete paralysis of the tail; 1.5, paresis of tail and mildly impaired righting reflex; 2.0, gait ataxia and severely reduced righting reflex; 2.5, bilateral severe hind limb paresis; 3.0, complete bilateral hind limb paralysis; 3.5, complete bilateral hind limb paralysis and forelimb paresis; 4, hind and fore limb paralysis; 5, moribund state or death. Mice received i.p. injections with Atrosab or Atrosimab on the days of EAE as stated. Control mice were injected with the appropriate species-specific control protein Fc∆ab.

### Joint Histopathology

The ankle joints were dissected, the skin was removed, and tissue was subsequently fixed in 4% aqueous formaldehyde solution overnight at RT, demineralized in EDTA decalcification solution (13% EDTA in 0.1M sodium phosphate buffer) at RT for 30 days and further placed in PBS at 4°C until further processing. Decalcified ankle joints were paraffin embedded in the sagittal plane. Paraffin blocks were sectioned using a microtome into 4 μm thick sections. The sections were then stained with H&E and evaluated by light microscopy for histopathological hallmarks of arthritis. The evaluation process was performed in a blinded fashion. Histopathological score (H score) was assessed based on the following scoring system: 0, no detectable pathology; 1, hyperplasia of the synovial membrane and presence of polymorphonuclear infiltrates; 2, pannus and fibrous tissue formation and focal subchondrial bone erosion; 3, cartilage destruction and bone erosion; 4, extensive cartilage destruction and bone erosion, bone outline structure is lost. Group means were calculated from the individual highest scores per joint in each mouse in the specific group.

### Liver Histopathology

For immunohistological analysis of liver tissue, the organ was embedded in paraffin and sections were prepared and analyzed as described (18). Briefly, Oli Red O staining was performed according to the manufacturer’s protocol (Sigma-Aldrich, St. Louis, MO, USA) and fibrosis was detected by Sirius Red staining according to the manufacturer’s protocol (Sigma-Aldrich) Four microscopic fields at a 100-fold magnification were used for quantification. For detection of activated caspase-3 liver sections were incubated with an antibody against activated caspase-3 (Cell Signaling, Danvers, MA, USA) for one hour following antigen retrieval and blocking of endogenous peroxidase. After repeated washings, sections were treated with biotinylated goat anti-rabbit antibody for 30 min and then covered with avidin-biotin complex reagent containing horseradish peroxidase (VECTASTAIN ABC Kit, Vector Laboratories, Burlingame, CA, USA) for one hour. The percentage of cells positive for activated caspase-3 was assessed by analyzing four microscopic fields at a 400-fold magnification.

### Spinal Cord Histopathology

Spinal cord histopathology was performed as described previously (11, 17). Briefly, mice were transcardially perfused with 4% PFA in PBS after an overdose of ketamine/xylazine. Spinal cords were isolated, processed for paraffin-embedding and 0.5 μm transverse sections were cut into serial sections. LFB staining was performed in order to assess demyelination. The degree of demyelination was evaluated semiquantitatively using the following scoring system: 0.5, traces of perivascular or subpial demyelination; 1, marked perivascular or subpial demyelination; 2, confluent perivascular or subpial demyelination; 3, demyelination of half spinal cord cross section; and 4, transverse myelitis. For immunohistochemistry, antigen retrieval was performed by incubating tissue sections in heated (~80°C) 0.2% citrate buffer (pH 6.0) for 15 mins, before being left to cool. An antibody against amyloid precursor protein (APP) (1:2500, Millipore, Darmstadt, Germany) was used to detect damaged axons. A minimum of 10 sections taken throughout the length of the spinal cord were quantified. All microscopy was performed on an Eclipse 80i upright microscope with 2x or 40x objectives and fitted with a DXM1200C camera (Nikon, Shinagawa, Tokyo, Japan).

### Statistics

Data was assessed for normality using the Shapiro-Wilk Test, followed by either a Mann-Whitney or a two-tailed student’s t-test for comparing two experimental groups, or the one-way analysis of variance (ANOVA) with post-hoc Bonferroni, Dunnett’s or Tukey’s test, or Kruskal-Wallis test with *post-hoc* Dunn’s analysis for multiple group comparisons. A p value of <0.05 was considered to be statistically significant.

## Results

### Biochemical Characterization and Bioactivity of Atrosimab

**Figure 1:**
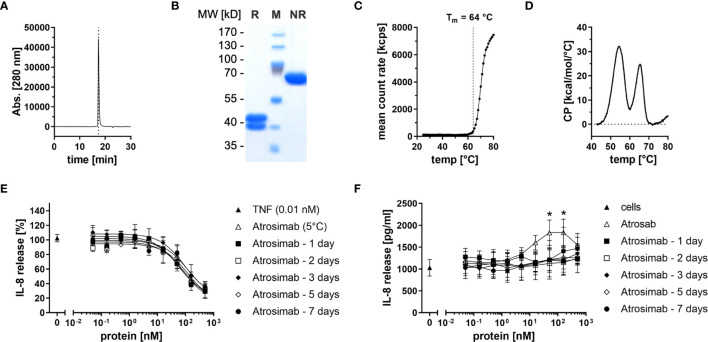
Biochemical and functional characterization of Atrosimab. **(A)** Atrosimab was analyzed by size exclusion chromatography (SEC) using a TSKgel SuperSW mAb high resolution column. **(B)** Atrosimab was resolved by SDS-PAGE under reducing (R) and non-reducing (NR) conditions and protein was visualized using Coomassie Brilliant Blue staining (M = markers). **(C, D)** Thermal stability was analyzed by **(C)** dynamic light scattering and **(D)** dynamic scanning calorimetry. **(E, F)** Atrosimab stored for the indicated times in 50% human serum at 37°C was added to HT1080 cells followed by addition of **(E)** 0.01 nM TNF or **(F)** without TNF. Atrosab stored at 5°C was used as a positive control. After 16-20 hours incubation at 37°C, 5% CO_2_, the supernatants were analyzed for secreted IL-8 by ELISA. Mean ± SD, n=3, Atrosab vs Atrosimab 1 day: *p < 0.05.

Atrosimab was produced in lentiviral transfected CHO cells and purified by protein A and CHT mixed-mode chromatography. Size-exclusion chromatography (SEC) and SDS-PAGE analysis using Coomassie staining revealed correct assembly and integrity of the heterodimeric protein comprising a VL1C chain (38 kDa) and a VHκC chain (43 kDa) (**Figures 1A, B**). Using dynamic light scattering (DLS), an aggregation temperature of 64°C was confirmed (19) (**Figure 1C**). By dynamic scanning calorimetry (DSC), two melting temperatures of 54°C and 65°C were determined (**Figure 1D**). We then determined long-term stability of the Atrosimab batch after storage at 5°C or 25°C, 60% relative humidity (RH). To determine antagonistic and potential agonistic activity of Atrosimab, we applied an IL-8 release assay using HT1080 cells in absence (agonist assay) and presence (antagonist assay) of 0.01 nM TNF. All tested Atrosimab batches showed a dose-dependent inhibition of the TNF-induced IL-8 release with IC_50_ values ranging between 21.9 nM and 34.9 nM without statistically significant differences. No major differences were observed between batches stored up to 9 months at 5°C or 25°C/60%RH (**Supplementary Figure 1A**). We next determined that storage of Atrosimab at 5°C and 25°C/60%RH did not result in formation of agonistic Atrosimab species (**Supplementary Figure 1B**). We then analyzed whether incubation of Atrosimab in human serum at 37°C impacts the blocking activity of Atrosimab or promotes formation of agonistic Atrosimab aggregates. Whereas the inhibitory activity of Atrosimab was 2-fold reduced after 7 days incubation (**Figure 1E**), incubation for up to 7 days in human serum at 37°C did not result in a detectable agonistic activity of Atrosimab (**Figure 1F**). Altogether, this indicates the high stability of Atrosimab and its suitability for clinical use as a TNFR1 antagonist.

### Species-Specific Binding and Epitope Mapping of Atrosimab

Using ELISA, Atrosimab was observed to bind to human, rhesus, and cynomolgus TNFR1 with similar EC_50_ values (2.2 nM for human TNFR1, 5.1 nM for rhesus TNFR1, and 1.9 nM for cynomolgus TNFR1). No binding of Atrosimab to mouse and rat TNFR1 was observed (**Supplementary Figure 2A**). As expected, Atrosimab did not bind to human or mouse TNFR2. The TNFR2 agonist EHD2-sc-mTNF_R2_ (20) was used as a positive control for TNFR2 binding (**Supplementary Figure 2B**). SPR measurements revealed comparable association and dissociation rates and a similar affinity of Atrosimab to human and cynomolgus TNFR1, with K*_D_* values of 5.01 nM and 3.48 nM, respectively (**Supplementary Figure 2C** and **Supplementary Table 1**). Thus, non-human primates are suitable species for further preclinical testing.

To further map the epitope recognized by Atrosimab, a set of 24 TNFR1 mutants was studied by ELISA. For this, the selectivity of the investigated proteins for human TNFR1 over mouse TNFR1 was exploited using human TNFR1 variants carrying murine residues at selected positions as single or double amino acid replacements (14). While human TNF was able to bind to all analyzed TNFR1 mutants (**Supplementary Figure 3A**), Atrosimab showed a reduced binding to the Q24K variant and a complete lack of binding to mouse TNFR1 as well as the human TNFR1 mutants P23S, L67A, R68A, and H69Q (**Supplementary Figure 3B**). Atrosab did not bind to mouse TNFR1 and the human TNFR1 mutants P23S, R68A and H69Q. However, Atrosab was still able to interact with variant L67A, while binding to L71A, which does not affect binding of Atrosimab, was strongly reduced (**Supplementary Figure 3C**). This finding indicates that affinity maturation led to alterations in interacting residues while maintaining the overall epitope region in TNFR1 (**Supplementary Figure 3D**).

### Pharmacokinetics of Atrosimab and Atrosab

**Figure 2:**
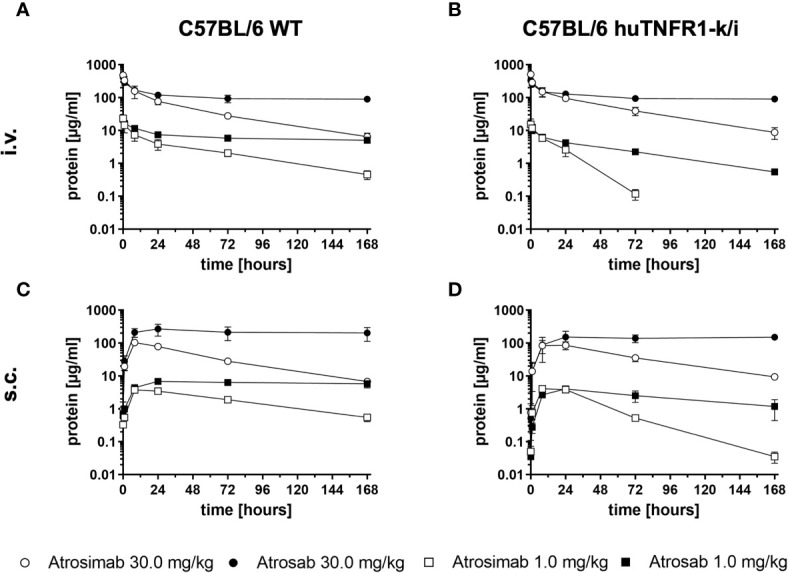
Comparative pharmacokinetic analysis of Atrosimab and ATROSAB. Atrosimab or Atrosab were injected **(A, B)** intravenously (*i.v.*) or **(C, D)** subcutaneously (*s.c.*) at doses of 1.0 mg/kg or 30.0 mg/kg body weight into **(A, C)** C57BL/6 wildtype (WT) or **(B, D)** C57BL/6 huTNFR1-k/i mice. Serum samples, collected at the indicated time points, were analyzed for remaining circulating protein by ELISA. Shown are the mean ± SD of three mice.

Pharmacokinetic analyses of Atrosimab in comparison to Atrosab were performed in C57BL/6 wild-type and C57BL/6 huTNFR1-k/i mice after intravenous (*i.v.)* or subcutaneous (*s.c.*) injections using doses of 1.0 mg/kg and 30.0 mg/kg body weight, respectively (**Figures 2A–D** and **Table 1**). Atrosimab showed similar terminal half-lives in C57BL/6 wild-type and C57BL/6 huTNFR1-k/i mice after i.v. injection at a dose of 30 mg/kg (approx. 32 h), while at 1 mg/kg terminal half-life was reduced from 31 h in C57BL/6 to 9.5 h in huTNFR1-k/i mice. Similar effects were observed after s.c. injections, indicative of target-mediated clearance effects in C57BL/6 huTNFR1-k/i mice. Generally, Atrosimab exhibited shorter half-lives under all tested conditions in comparison to Atrosab. Thus, Atrosab showed at the higher dose an approximately 6.8-fold prolonged half-life compared to Atrosimab after i.v. injection into C57BL/6 wild-type mice and approximately 12-fold in C57BL/6 huTNFR1-k/i mice. This reduced half-life of Atrosimab is presumably due to the lower molecular mass compared to Atrosab (78 kDa *vs* 150 kDa).

**Table 1:** Pharmacokinetic analysis of Atrosimab and Atrosab in huTNFR1-k/i and wildtype mice.

Mice	huTNFR1-k/i		wild type	
Protein	Atrosimab	Atrosab	Atrosimab	Atrosab
Dose - Route	30.0 mg/kg - i.v.		30.0 mg/kg - i.v.	
Parameter	Mean ± SD	Mean ± SD	Mean ± SD	Mean ± SD
**t_1/2_Alpha (h)**	0.66 ± 0.14	1.27 ± 0.4	2.26 ± 2.03	1.36 ± 0.44
**t_1/2_Beta (h)**	31.52 ± 4.63	380.36 ± 315.34	32.77 ± 7.54	225.18 ± 44.97
**AUC 0-t (μg/ml × h)**	7983.8 ± 893.4	18548.2 ± 1230.3	6989.7 ± 1354	18895.8 ± 1835.4
**Dose - Route**	**1.0 mg/kg - i.v.**		**1.0 mg/kg - i.v.**	
**t_1/2_Alpha (h)**	5.41 ± 6.16	1.32 ± 1	1.05 ± 0.51	1.9 ± 1.6
**t_1/2_Beta (h)**	9.52 ± 5.94	45.69 ± 7.13	31.37 ± 7.09	211.82 ± 144.78
**AUC 0-t (μg/ml × h)**	177.8 ± 38.13	406.6 ± 36.78	365.78 ± 92.17	1124.37 ± 57.54
**Dose - Route**	**30.0 mg/kg - s.c.**		**30.0 mg/kg - s.c.**	
**t_1/2_Beta (h)**	41 ± 6.5	n.d.	33.7 ± 3.6	431.7 ± 188.7
**AUC 0-t (μg/ml × h)**	6816.7 ± 1334	23357.7 ± 5695.5	6190.7 ± 404.6	36393.3 ± 12331
**Dose - Route**	**1.0 mg/kg - s.c.**		**1.0 mg/kg - s.c.**	
**t_1/2_Beta (h)**	16.75 ± 0.71	78.52 ± 22.86	54.56 ± 4.57	927.93 ± 561.42
**AUC 0-t (μg/ml × h)**	214.07 ± 23.82	408.73 ± 117.62	321.83 ± 5.4	1018.93 ± 153.86

t1/2Alpha, initial half-life; t1/2beta, terminal half-life; AUC 0-t, area under the curve until the last detected time point.

### Tolerance and *In Vivo* Activity of Atrosimab After Systemic Application

**Figure 3:**
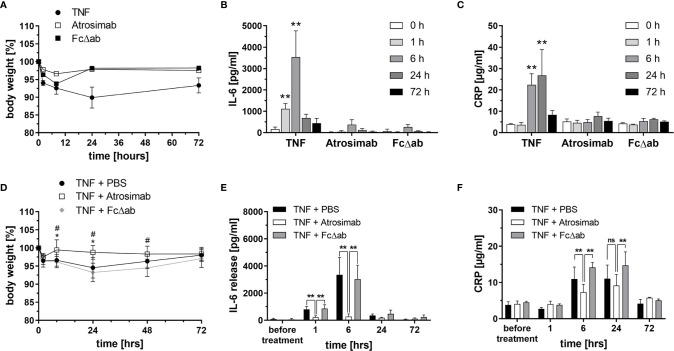
Atrosimab blocks acute TNF-induced inflammation *in vivo.* **(A–C)** C57BL/6 huTNFR1-k/i mice received *i.v.* injections of TNF (0.3 mg/kg), Atrosimab or FcΔab (30 mg/kg). **(A)** Body weight was determined and **(B, C)** blood samples were collected before treatment (0 h) and 1 h, 6 h, 24 h and 72 h after the injections. **(B)** IL-6 and **(C)** CRP levels in the serum were determined by ELISA. **(D–F)** C57BL/6 huTNFR1-k/i mice received *i.v.* injections of PBS, Atrosimab (30 mg/kg body weight) or FcΔab (30 mg/kg). TNF (0.3 mg/kg) was injected 30 minutes thereafter to induce acute inflammatory responses. **(D)** Body weight was determined and **(E, F)** blood samples were collected before treatment (0 h) and 1 h, 6 h, 24 h and 72 h after TNF injection. **(E)** IL-6 and **(F)** CRP levels in the serum were determined by ELISA. Individual values were excluded using the ROUT method of outlier identification or because they were below the detection limit (Q = 1%) or because they were below the detection limit of the ELISA. Mean ± SD, n=6. **(A, C)**: **p < 0.01; **(D)**: *p < 0.05 – TNF + PBS *vs* TNF + Atrosimab, ^#^p < 0.05 – TNF + PBS *vs* TNF + FcΔab; **(E, F)**: ns, not significant, **p < 0.01.

Safety and tolerability of Atrosimab, injected *i.v.* at a dose of 30 mg/kg body weight, was analyzed in huTNFR1-k/i mice. An Fc region of IgG1, carrying the same mutations as Atrosimab that silence Fc effector functions (24) was used as a target-independent control protein (FcΔab). As a positive control for TNF receptor activation, TNF was injected at a sublethal dose of 0.3 mg/kg body weight. Only TNF injection induced a significant loss of body weight (**Figure 3A**) and a time-dependent increase in serum IL-6 (**Figure 3B**) and CRP (**Figure 3C**) levels. In contrast, both Atrosimab and FcΔab did not significantly alter body weight or induce elevated levels of the inflammatory proteins IL-6 and CRP (**Figures 3A–C**). Application of both molecules induced a slight but not significant elevation of IL-6 6 hours after injection (**Figure 3B**). In summary, Atrosimab could be safely applied at the indicated dose.

Next, we analyzed whether the same dose blocks TNF-induced release of the inflammatory cytokine IL-6 *in vivo*. For that, mice received i.v. injections of PBS, Atrosimab or FcΔab as a prophylactic treatment, followed by an i.v. TNF (0.3 mg/kg) injection after 30 minutes to induce a TNF-mediated inflammatory response. PBS- and FcΔab-treated groups showed a TNF-induced reduction of body weight, lasting for 48 hours post injection (**Figure 3D**) and increased IL-6 serum levels up to 24 hours post injection (**Figure 3E**). In contrast, Atrosimab completely blocked the TNF-induced weight loss (**Figure 3D**) and significantly reduced the serum IL-6 levels at the 1 hour and 6 hours data points to almost baseline levels (**Figure 3E**). Similar, compared to the control protein Fc∆ab, Atrosimab significantly reduced TNF-induced increase in systemic CRP levels after 6 hours (**Figure 3F**). This indicates that Atrosimab potently blocks TNF-induced inflammatory responses *in vivo*.

### Atrosimab Is Therapeutic in a Model of Experimental Arthritis

**Figure 4:**
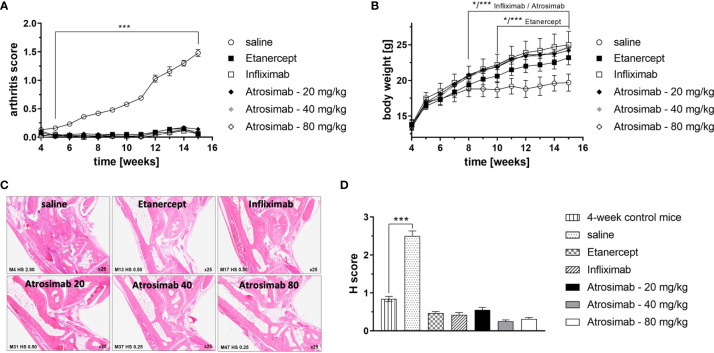
Atrosimab prevents development of arthritic disease. Tg197hTNFR1KI transgenic mice were used as a model of experimental arthritis. Treatment (s.c.) with saline, the anti-TNF therapeutics Etanercept or Infliximab (20 mg/kg each), or different doses of Atrosimab was started at 4 weeks of age and repeated twice weekly. Arthritis score **(A)** and weight **(B)** were documented weekly. **(C, D)** Histopathological analysis of the joints was performed at an age of 15 weeks and compared to 4-week-old non-treated mice. Shown are **(C)** representative pictures and **(D)** quantification of the histological (H) score. Mean ± SD, n=8, *p < 0.05, ***p < 0.001.

Anti-TNF therapeutics are approved for the treatment of rheumatoid arthritis. We therefore first compared the therapeutic activity of Atrosimab to anti-TNF drugs using a model of experimental arthritis using Tg197hTNFR1KI transgenic mice. The double transgenic Tg197hTNFR1KI mice therefore offer the possibility to compare the therapeutic activity of Atrosimab with conventional anti-human TNF drugs. We first analyzed different Atrosimab doses (20, 40 and 80 mg/kg) in a prophylactic setting by starting injections at 4 weeks of age, before onset of arthritic disease. All Atrosimab doses and the anti-TNF therapeutics Etanercept and Infliximab prevented development of arthritic disease (**Figure 4A**) and disease-associated inhibition of body weight gain (**Figure 4B**). In contrast to saline treated mice, none of the therapeutics-treated mice showed histopathological changes beyond the level seen for 4-week-old control mice (**Figures 4C, D**). This indicated that Atrosimab, similar to previously documented anti-TNF therapeutics, also suppresses disease development in a chronic inflammatory disease.

**Figure 5:**
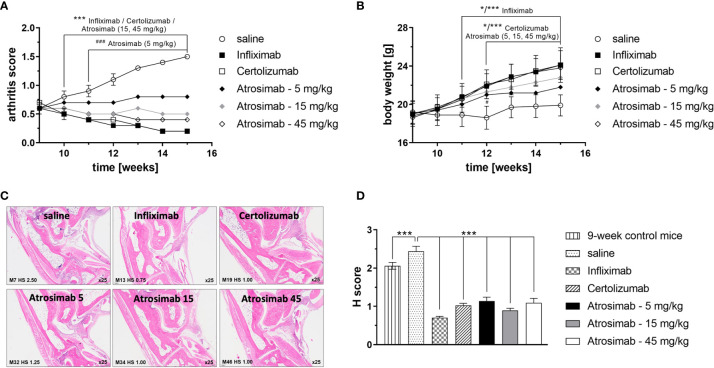
Atrosimab is therapeutic for arthritic disease. Tg197hTNFR1KI transgenic mice were treated at an age of 9 weeks with saline, the anti-TNF therapeutics Infliximab or Certolizumab pegol (15 mg/kg each), or different doses of Atrosimab by twice weekly s.c. injections. Arthritis score **(A)** and weight **(B)** were documented weekly. **(C, D)** Histopathological analysis of the joints was performed at an age of 15 weeks and compared to 9-week-old non-treated mice. Shown are representative pictures and quantification of the histological (H) score. Mean ± SD, n=8, *p < 0.05, ***/^###^p < 0.001.

We next analyzed the therapeutic efficacy of Atrosimab in a therapeutic setting by treating mice with established arthritis. Therefore, treatment with Atrosimab (5, 15 and 45 mg/kg) was delayed until week 9 of age, when mice had developed a profound arthritic score. Atrosimab treatment was compared to the anti-TNF therapeutics Infliximab and Certolizumab pegol. All therapeutics alleviated arthritic disease (**Figures 5A, B**). Here, a dose-dependent therapeutic efficacy of Atrosimab was observed. Whereas the low dose Atrosimab treatment (5 mg/kg) halted disease progression, increased Atrosimab doses showed an improved therapeutic activity. The higher Atrosimab doses showed an efficacy comparable to the anti-TNF therapeutics Infliximab and Certolizumab pegol. Application of all therapeutics resulted in an improved histopathological analysis (**Figures 5C, D**). Here, no dose-dependent effects were observed for Atrosimab-treated mice. Notably, all treatment groups showed a H score below the level of 9-week-old control animals, indicating the histopathological improvement after therapy.

### Atrosimab Alleviates Fibroses in a Model of Non-Alcoholic Fatty Liver Disease

**Figure 6:**
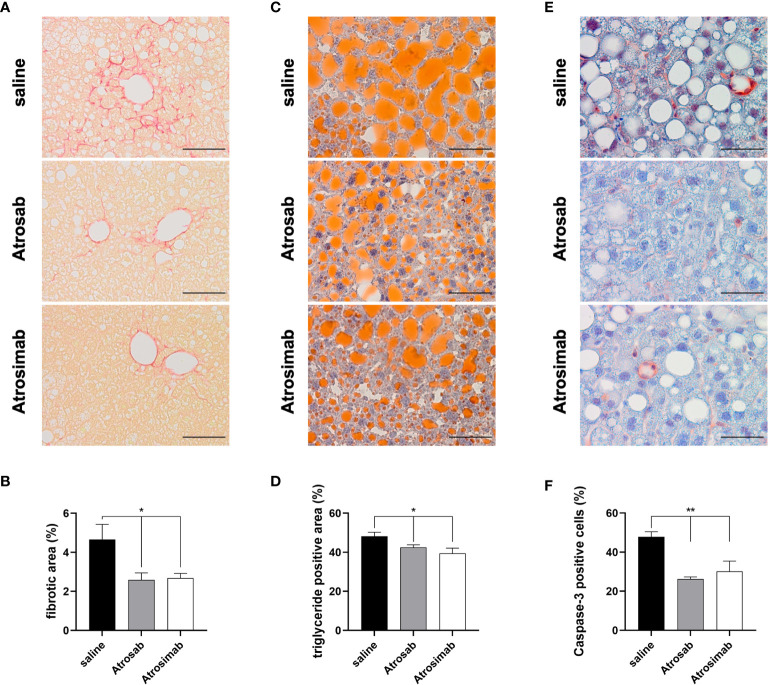
Atrosimab alleviated fibrosis, steatosis and liver injury in a model of NAFLD. huTNFR1-k/i mice were fed a high-fat diet for 21 weeks and then treated with saline, Atrosimab and Atrosab for 8 weeks. Liver pathology was analyzed by quantifying **(A, B)** liver fibrosis, **(C, D)** steatosis and **(E, F)** liver injury. **(A, B)** Liver fibrosis was quantified using Sirius Red staining, **(C, D)** liver steatosis using Oil Red O staining to identify triglyceride content and **(E, F)** liver injury by detection of caspase-3 activity. Shown are representative pictures **(A, C, E)** and quantification of the histological analyses (B, D, F). a: scale bars = 100 µm, c,e: scale bars = 50 µm. Mean ± SEM, **(B)**: n=9 saline, n=8 Atrosab, n=7 Atrosimab; **(D)**: n=9 saline, n=6 Atrosab, n=5 Atrosimab, **(F)**: n=5 each: *p < 0.05, **p < 0.01.

Recently, a clinical study showed that there are no beneficial effects of anti-TNF agent use for development of cirrhosis, non-alcoholic fatty liver disease (NAFLD) or NASH in patients with immune-related diseases (25). However, we have previously shown that the TNFR1 inhibitor Atrosab reduces liver steatosis, hepatocellular injury and fibrosis in a mouse model of NAFLD (18). We therefore compared the therapeutic activity of Atrosimab to Atrosab using the NASH model. To induce steatohepatitis, we fed huTNFR1-k/i mice with a high-fat diet (HFD) for 21 weeks and then treated the NASH mice by biweekly injection with saline, Atrosab or Atrosimab (45 mg/kg each). After 8 weeks, liver pathology was analyzed by immunohistology. NASH-induced apoptotic liver injury triggers fibrosis, a major factor of mortality in human NAFLD patients and an important end point in clinical NAFLD trials. Both Atrosab and Atrosimab treatment significantly reduced liver fibrosis, assessed by Sirius Red staining (**Figures 6A, B**). Similar, both Atrosab and Atrosimab resulted in reduced liver steatosis, detected by Oil Red O staining (**Figures 6C, D**). Last, we analyzed the impact of TNFR1 antagonist treatment on apoptotic liver injury by immunohistochemical detection of activated caspase-3. In line with the reduction of liver fibrosis and steatosis, caspase-3 activity was decreased in liver tissues from Atrosab- and Atrosimab-treated NASH mice, compared to saline-treated mice (**Figures 6E, F**). Altogether, these data indicate that Atrosimab shows a comparable therapeutic activity to Atrosab, despite its shorter half-life and monovalent TNFR1 binding.

### Atrosimab Ameliorates Experimental Autoimmune Encephalomyelitis

**Figure 7:**
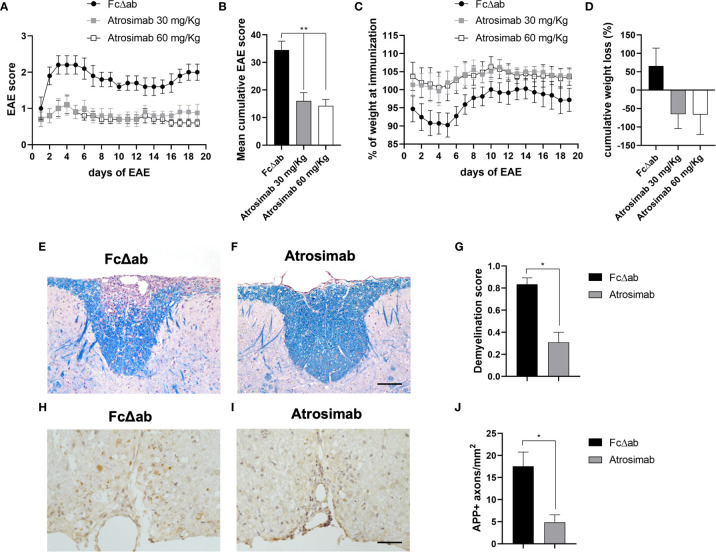
Atrosimab ameliorates disease in a model of multiple sclerosis. huTNFR1-k/i mice were immunized with MOG^35-55^ peptide to induce EAE. Mice were injected i.p. on days 1, 4, 8 and 12 of manifest disease with either 30 mg/kg or 60 mg/kg Atrosimab, or 30 mg/kg FcΔab. Both dosages of Atrosimab led to an immediate and sustained improvement in **(A)** motor symptoms, reflected by the **(B)** cumulative EAE score, as well as a reduction in **(C)** disease-associated weight loss, also supported by **(D)** the cumulative weight loss. Two of the major pathological hallmarks of EAE, demyelination and axonal degeneration were investigated histopathologically. **(E–G)** Luxol fast blue (LFB) staining revealed, in comparison to FcΔab treated animals, significantly reduced demyelination following treatment with 30 mg/kg Atrosimab. **(H–J)** Treatment with 30 mg/kg Atrosimab also lead to a reduction in the number of damaged axons indicated by APP accumulation. Representative images are shown from Fc control **(E, H)** and Atrosimab-treated **(F, I)** mice. e,f,h,i: scale bars = 50 µm. a-d: Mean ± SEM, n=6; g,j: n=6 per group: *p<0.05, **p<0.01.

Whereas anti-TNF drugs failed in a clinical MS trial, selective blocking of TNFR1 was therapeutic in the EAE model of MS (11, 17). Therefore, we next validated the therapeutic activity of Atrosimab in EAE mice. We treated mice after onset of motor disease using a treatment regimen that was used for preclinical evaluation of Atrosab in EAE mice before (17). Atrosimab at a dose of 30 and 60 mg/kg ameliorated EAE motor disease compared to treatment with Fc∆ab (**Figures 7A, B**) and prevented disease-associated weight loss (**Figures 7C, D**). No differences were observed between the two Atrosimab doses, indicating that a dose of 30 mg/kg is sufficient to induce the maximal therapeutic activity of Atrosimab in this model. Histopathological analysis of the spinal cord demonstrated that treatment with 30 mg/kg resulted in significant reduction of demyelination, assessed with LFB staining (**Figures 7E–G**). Further, we observed a significant reduction of APP accumulation after Atrosimab injections (**Figures 7H–J**), indicating reduced axonal injury after Atrosimab treatment.

Our published data indicate that anti-TNFR1 therapy restricts immune cell infiltration across the blood-brain barrier through the downregulation of TNF-induced adhesion molecules resulting in ameliorated neuronal injury and EAE motor disease (17). We therefore confirmed that blocking of TNFR1 with Atrosimab similarly reduced TNF-mediated upregulation of adhesion proteins ICAM-1 and VCAM-1 using the human-derived endothelial cell line hCMEC/D3 (21) (**Supplementary Figure 4**).

## Discussion

Bivalent binding of TNFR1 antagonists harbors the risk for dose-dependent activation of TNFR1 and therefore renders bivalent TNFR1 antagonists, such as Atrosab, unsuitable for clinical use (10, 19, 26). In this study, we validated the therapeutic efficacy of the novel monovalent TNFR1 antagonist Atrosimab (19) in different models of chronic inflammation. Our data demonstrate that TNFR1 inhibition through Atrosimab administration efficiently blocked TNFR1-mediated inflammatory responses in a model of acute TNF-mediated inflammation and in experimental arthritis, NASH and EAE. This is in accordance with published data by us and others showing that TNFR1 inhibition is therapeutic for EAE (11, 17, 27–29), NASH (18) and experimental arthritis (13).

We previously demonstrated that the epitope of Atrosab is located at the ligand-binding site of the receptor (14). Epitope fine mapping confirmed that as expected Atrosimab and Atrosab share the same binding region. However, in contrast to Atrosab, position L67 was also important for binding of Atrosimab to TNFR1. This difference is most likely a result of the affinity maturation and re-engineering of the humanized variable domains in Atrosimab that also lead to improved affinity of Atrosimab to TNFR1 compared to Atrosab (19). Most importantly, we could demonstrate that Atrosimab does not form agonistic molecular species after long-term storage at 5°C or 25°C/60%RH as well as in human serum stored at 37°C. Accordingly, no agonistic activity of Atrosimab was observed after application into mice. In contrast, injection of Atrosab resulted in low but increased circulating cytokine levels due to activation of TNFR1 (data not shown). Altogether these data indicate that Atrosimab is well tolerated and a suitable drug candidate for further preclinical and clinical evaluation as TNFR1 antagonist.

Anti-TNF drugs have a long-standing clinical history and are among the best-selling biologics worldwide (4, 10, 30). Despite the clinical success of anti-TNF-therapeutics, there are, however, some setbacks. In particular, black box warnings were issued for anti-TNF drugs, including an increased risk to develop serious infections (31). The clinical use of anti-TNF therapeutics is further limited by failed clinical trials in MS patients. In particular, a phase II randomized, multi-center, placebo-controlled study using the anti-TNF Lenercept had to be stopped after Lenercept-treated patients showed a dose-dependent increase in exacerbations and neurological deficits compared to patients receiving placebo (7). A similar outcome of anti-TNF therapy in MS patients was observed in an open-label phase I safety trial where infliximab treatment induced increased MRI activity and immune activation in two rapidly progressive MS patients (6). Furthermore, some juvenile rheumatoid arthritis patients developed MS-like demyelination during anti-TNF therapy (8). Altogether these data indicate that neutralization of the TNF ligand is contraindicative in neurological diseases. Our and other data indicate that these limitations are dependent on inhibition of neuroprotective TNFR2 signaling (10, 22, 32–35) by conventional anti-TNF therapeutics. Indeed, genome-wide association studies identified a single nucleotide polymorphism (SNP) in the TNFRSF1A gene coding for TNFR1 that is associated with MS but not with other autoimmune diseases, where anti-TNF therapy is working. This SNP results in expression of a soluble TNFR1 that can bind and neutralize TNF comparable to anti-TNF therapeutics (36). Therefore, anti-TNFR1 therapeutics hold great promise as effective treatments for inflammatory diseases of the nervous system like MS, because they efficiently block pro-inflammatory signaling *via* TNFR1 while leaving regenerative and immunomodulatory TNFR2 signaling intact (10). Our data indicate that Atrosimab given at the onset of EAE disease halts disease progression after the first injection. A similar therapeutic efficacy was observed after Atrosab treatment of EAE mice (17). Interestingly, anti-TNF therapeutics do not only alter TNF activity, but also impact tmTNF reverse signaling. Indeed, it was shown that anti-TNF drugs induce anti-inflammatory responses through tmTNF reverse signaling *in vitro* and *in vivo* (37, 38). We do not expect Atrosimab to interfere with tmTNF reverse signaling.

Using the NASH model of NAFLD, we also observed comparable therapeutic activities of Atrosab and Atrosimab. This is particularly important since specific drugs for NAFLD are unavailable, and the therapeutic possibilities are limited and restricted to lifestyle intervention so far. Our observation that Atrosimab reduces liver injury, steatosis and fibrosis is of clinical relevance since NASH is histologically characterized by the presence of liver steatosis and evidence of liver injury which can result in the development of liver fibrosis/cirrhosis (39). Our results together with evidence from the literature that TNFR1 contributes to fibrotic liver injury (18), insulin resistance (40) and hepatocellular carcinoma formation in NAFLD (41, 42) indicate that TNFR1 is a promising novel drug target for NAFLD and highlight the potential of Atrosimab as a novel therapeutic to treat NAFLD. Notably, we determined a shorter half-life of Atrosimab compared to the full IgG Atrosab. The fact that Atrosimab induces similar therapeutic effects compared to Atrosab in EAE and NASH, indicates that the improved affinity of Atrosimab (19) balances its limited half-life.

Similar results were observed in the experimental arthritis study. Here in a prophylactic setting, Atrosimab was as effective as the conventional anti-TNF therapeutics Etanercept and Infliximab. In a therapeutic treatment setting, Atrosimab was slightly less effective than the anti-TNF drugs Infliximab and Certolizumab when given at the same dose (15 mg/kg). This can be explained by the shorter half-life of the antibody derivative Atrosimab compared to the full antibody Infliximab or the PEGylated, and thus half-life extended, Fab Certolizumab pegol. This limitation can be circumvented by either increasing the dose or the application frequency of Atrosimab. In particular, Atrosimab therefore may be a suitable treatment for patients that lose their therapeutic response to conventional anti-TNF drugs, e.g. due to formation of anti-drug antibodies. Altogether, our data obtained in the arthritis and EAE model demonstrate that Atrosimab is a suitable drug candidate for the treatment of chronic inflammatory diseases.

In contrast to conventional anti-TNF drugs, Atrosimab not only blocks TNF signaling *via* TNFR1, but also inhibits lymphotoxin α (LTα) signaling *via* TNFR1 (10). This may be of particular importance due to the pro-inflammatory role of LTα that was demonstrated in different mouse models of inflammatory diseases, including experimental arthritis and EAE (43–45). In particular, LTα was shown to exacerbate inflammation and demyelination in mice (46) and, like TNF, to be upregulated in cerebrospinal fluid mononuclear cells (47) and lesions (48) of MS patients. Similar to the situation in MS, elevated LTα levels were found in the synovium and serum RA patients (49, 50) and *in vitro* studies suggest that LTα induces proliferation of RA fibroblast-like synoviocytes (51). Altogether, these data suggest that LTα contributes to the pathology of inflammatory diseases. However, pateclizumab, an antagonistic anti-LTα antibody, was not as efficacious as the anti-TNF agent adalimumab in reducing symptoms of RA in a head-to-head study (51, 52). Notably, RA patients with resistance to infliximab treatment still responded to Etanercept, a TNFR1-Fc fusion protein that in contrast to Infliximab also neutralizes LTα (53). Therefore, in this regard Atrosimab may be superior to conventional anti-TNF antibodies due to its increased safety profile as well as neutralization of the pro-inflammatory TNF and LTα pathways.

In summary, we have demonstrated that Atrosimab is therapeutic in different models of acute and inflammatory diseases and provide necessary pre-clinical data to support further clinical development of Atrosimab as a novel drug candidate. Altogether our data confirm the high potential of the TNFR1 antagonists Atrosimab as a safe and effective novel drug to treat acute and chronic inflammatory diseases.

## Data Availability Statement

The original contributions presented in the study are included in the article/**Supplementary Material**. Further inquiries can be directed to the corresponding authors.

## Ethics Statement

The animal study was reviewed and approved by Regierungspräsidium Stuttgart, Regierungspräsidium Heidelberg and Regierungspräsidium Hannover. Written informed consent was obtained from the owners for the participation of their animals in this study.

## Author Contributions

FR, SW, KJ, CH, CV, HZ, RiF, KPi, SM, and AV conducted experiments. M-AD, SH, AH, KPf, HB, and RD designed parts of the experimental work, analyzed results, and contributed to writing the manuscript. RK and RoF designed and supervised the project, analyzed the results, and wrote the manuscript. All authors contributed to the article and approved the submitted version.

## Funding

This work was supported by a research grant from Baliopharm, Basel, CH.

## Conflict of Interest

Author M-AD, SH and AH were employed by company Baliopharm. FR, AH, KP and REK are named inventors on patents and patent applications covering the Atrosimab technology. 

The remaining authors declare that the research was conducted in the absence of any commercial or financial relationships that could be construed as a potential conflict of interest.

The authors declare that this study received funding from Baliopharm. The funder was involved in the design of the experimental arthritis study.
