# Supplementary figures and images for: The TNFR1 Antagonist Atrosimab Is Therapeutic in Mouse Models of Acute and Chronic Inflammation
Source: Front Immunol. 2021 Jul 7;12:705485. doi: 10.3389/fimmu.2021.705485 (PMC8294390; doi:10.3389/fimmu.2021.705485)

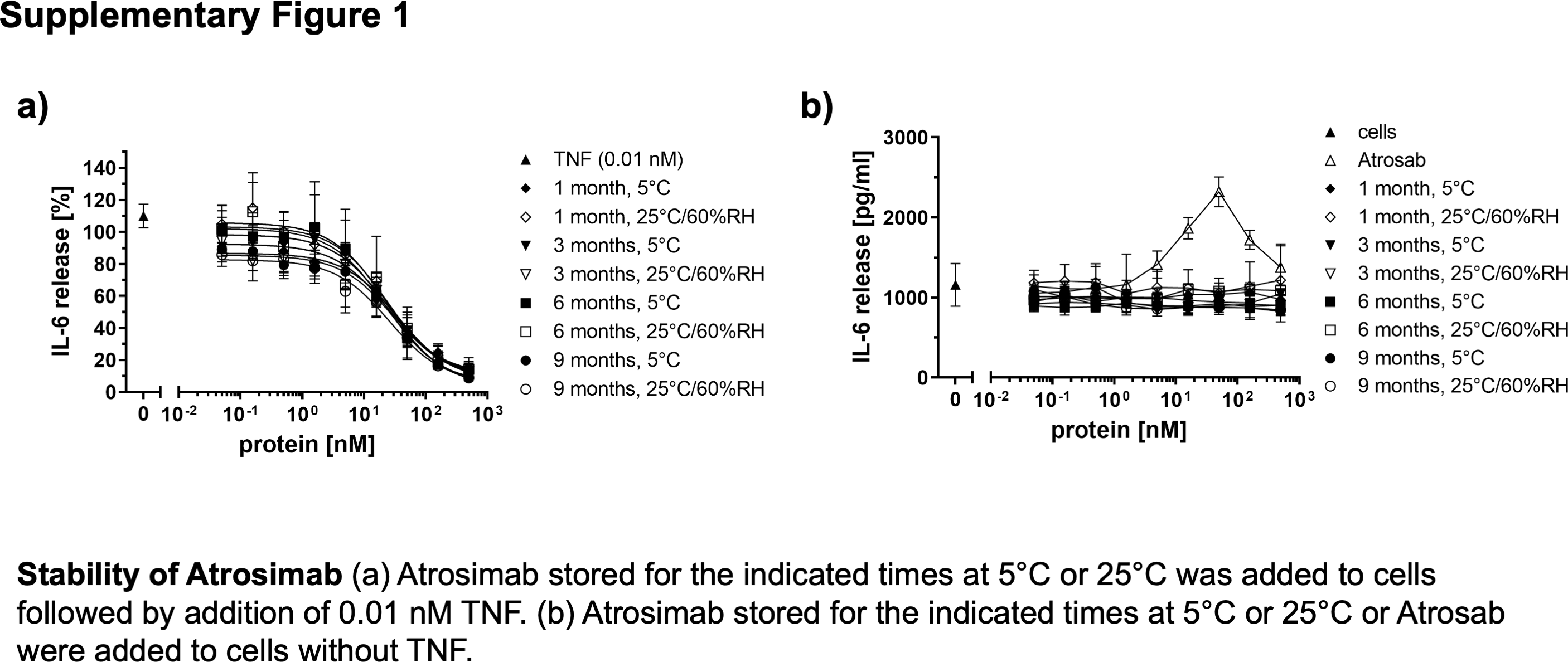


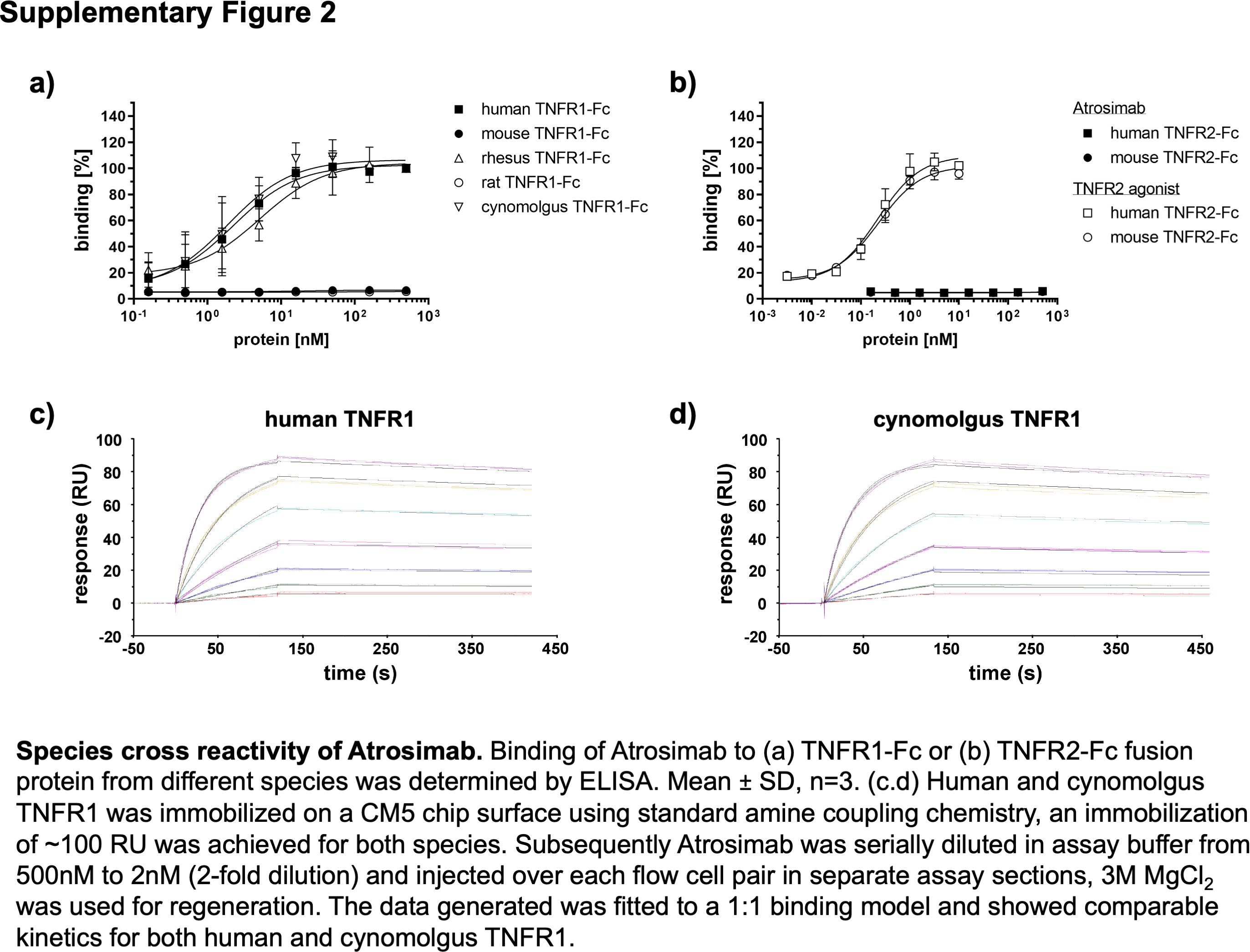


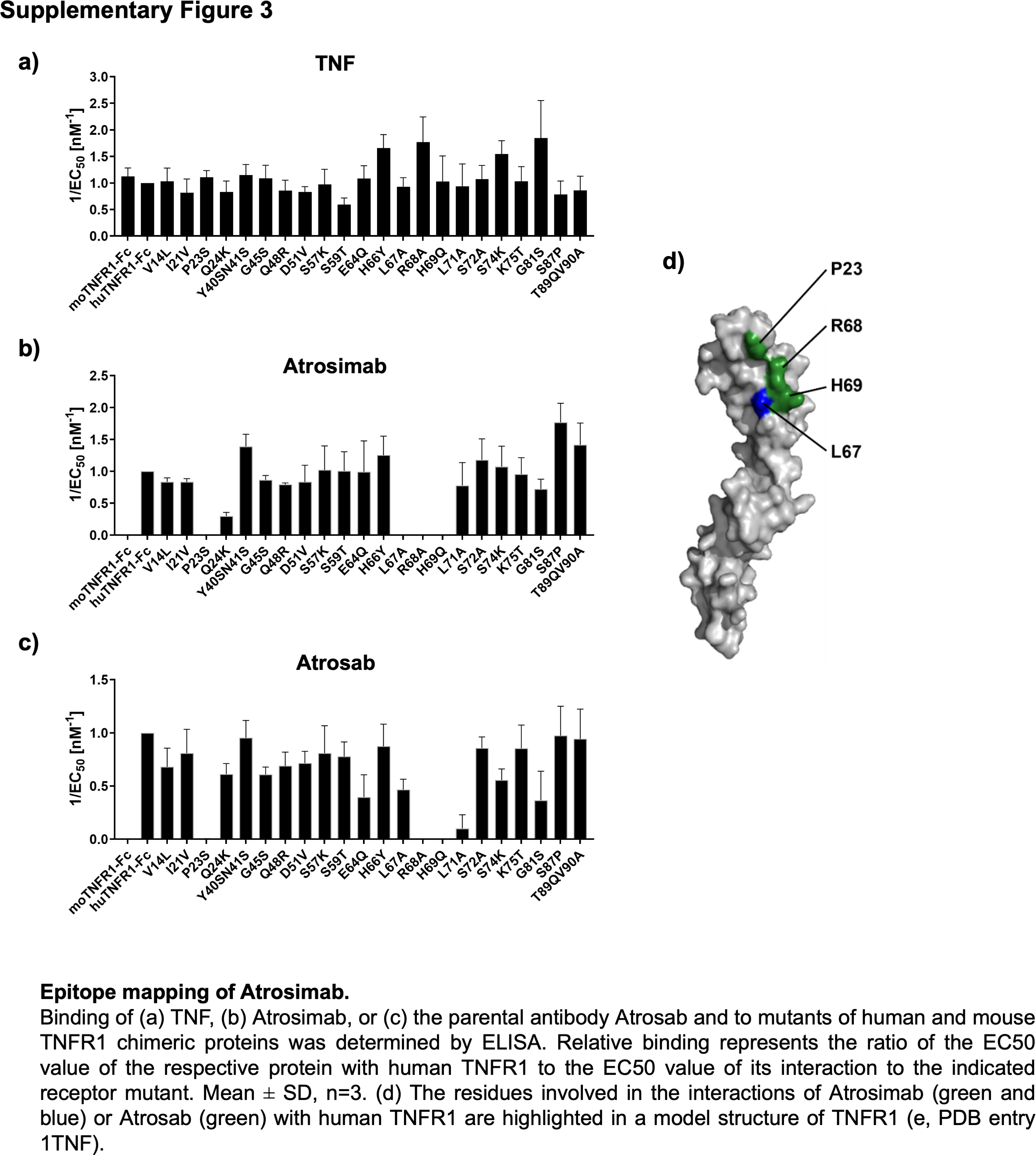


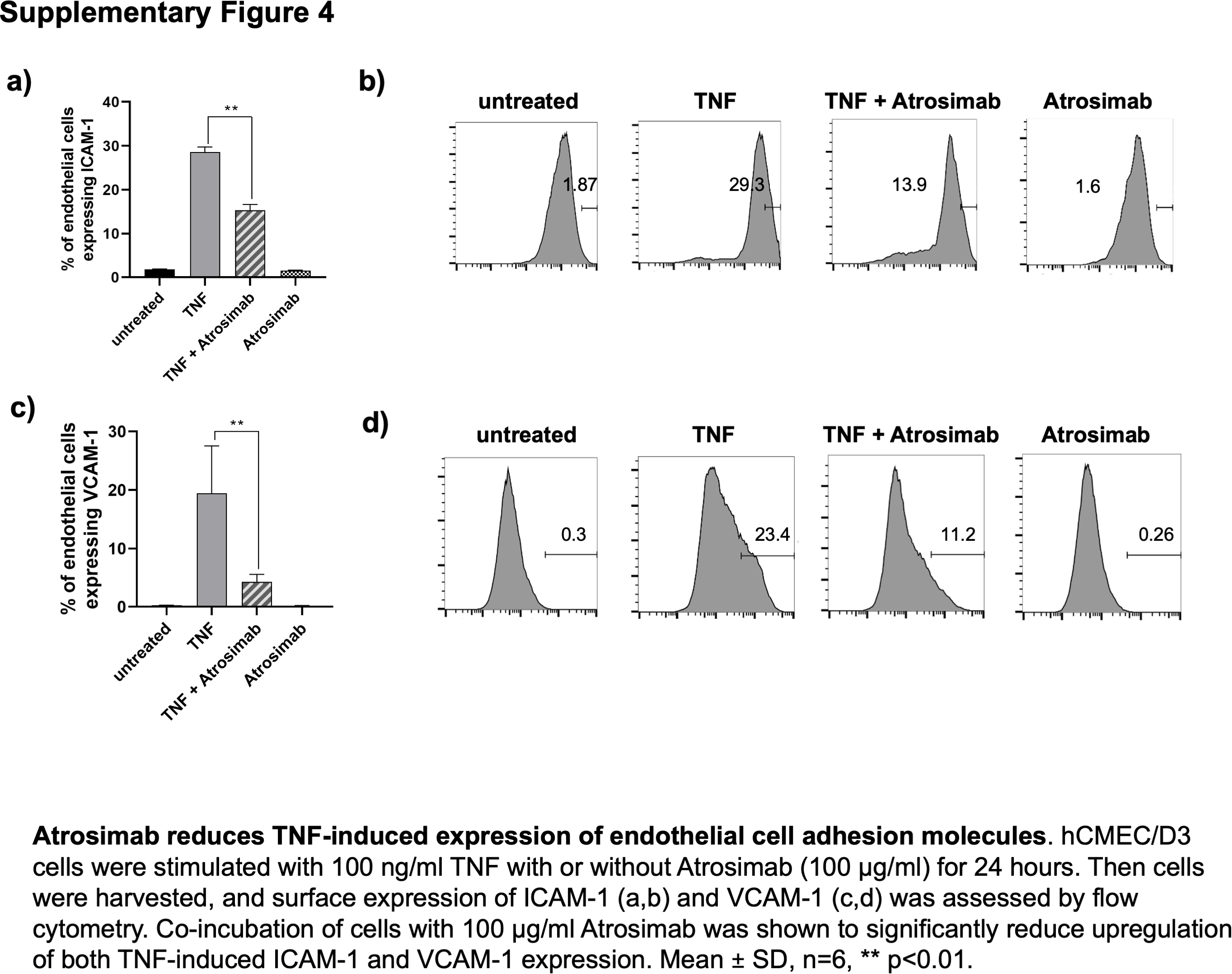


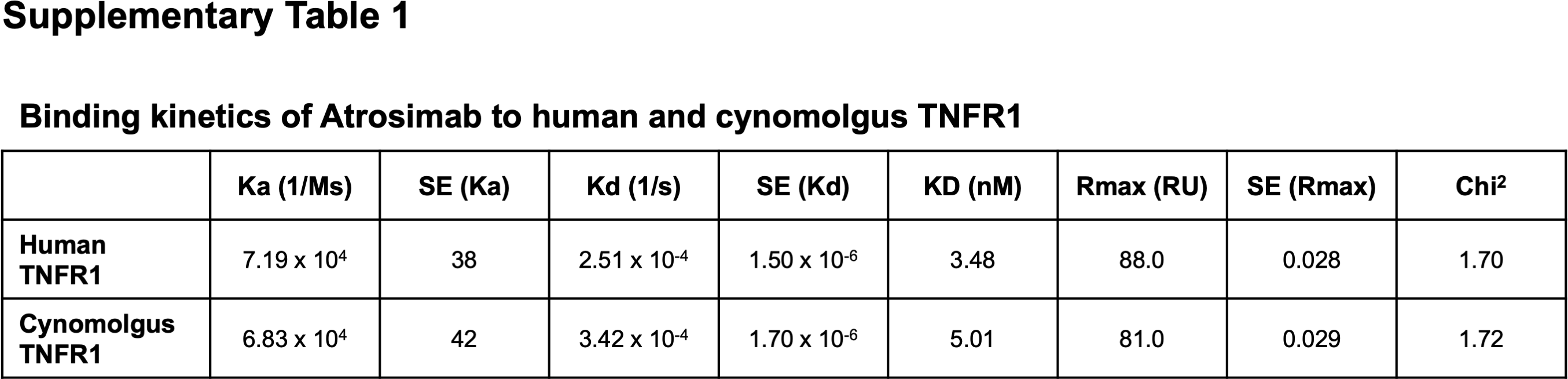

Supplement: Supplementary file 1 [file DataSheet_1.docx]
